# Dr. Simmons’ Record

**Published:** 1905-08

**Authors:** 


Dr. Simmons' Record.—I have said that Simmons was a homoeopath before he became editor of ye Octopus. I give my authority. The Chicago Medical Directory, published under the supervision of the Chicago Medical Society (1905) says (page 252): "Simmons, G. H., Hahnemann Medical College, 1882; Rush, 1892; Editor Journal of American Medical Association."

